# Aggregation Mechanism of Alzheimer’s Amyloid β-Peptide Mediated by α-Strand/α-Sheet Structure

**DOI:** 10.3390/ijms21031094

**Published:** 2020-02-07

**Authors:** Anand Balupuri, Kwang-Eun Choi, Nam Sook Kang

**Affiliations:** Graduate School of New Drug Discovery and Development, Chungnam National University, 99 Daehak-ro, Yuseong-gu, Daejeon 34134, Korea; balupuri@cnu.ac.kr (A.B.); hwendiv@naver.com (K.-E.C.)

**Keywords:** amyloid-β peptide, α-strand, α-sheet, MD simulation, Alzheimer’s disease

## Abstract

Alzheimer’s disease (AD) is one of the most common neurodegenerative diseases and a widespread form of dementia. Aggregated forms of the amyloid β-peptide (Aβ) are identified as a toxic species responsible for neuronal damage in AD. Extensive research has been conducted to reveal the aggregation mechanism of Aβ. However, the structure of pathological aggregates and the mechanism of aggregation are not well understood. Recently, experimental studies have confirmed that the α-sheet structure in Aβ drives aggregation and toxicity in AD. However, how the α-sheet structure is formed in Aβ and how it contributes to Aβ aggregation remains elusive. In the present study, molecular dynamics simulations suggest that Aβ adopts the α-strand conformation by peptide-plane flipping. Multiple α-strands interact through hydrogen bonding to form α-sheets. This structure acts as a nucleus that initiates and promotes aggregation and fibrillation of Aβ. Our findings are supported by previous experimental as well as theoretical studies. This study provides valuable structural insights for the design of anti-AD drugs exploiting the α-strand/α-sheet structure.

## 1. Introduction

Alzheimer’s disease (AD) is one of the most common neurodegenerative diseases and widespread form of dementia. AD is clinically characterized by progressive memory and neuronal loss combined with cognitive impairment. There is currently no approved treatment with a proven disease-modifying effect despite the decades of research. A worldwide effort is underway to find drugs that target the underlying pathology of AD [1,2]. According to the amyloid hypothesis, accumulation of amyloid β-peptide (Aβ peptide) in the brain is the main cause of AD [3,4]. Several point mutations in the Aβ sequence are linked to early-onset AD [5]. The amyloid hypothesis remains the dominant theoretical perspective in AD research and continues to influence the development of potential treatments [4,6]. Pharmaceutical companies have conducted extensive research on developing drugs that target Aβ in different ways. Transgenic mice are often used for studying AD. Although several Aβ-centric approaches worked well in mice, none have succeeded in clinical trials yet. It is reported that a number of these trials were misdesigned in terms of patient selection, choice of agent, target engagement, and dose [6]. Many therapeutics that were supposed to reduce Aβ production or aggregation have failed in Phase III clinical trials. However, several others are in various stages of development [7,8]. Tramiprosate, tarenflurbil, and semagacestat have been discontinued [7]. The γ-secretase inhibitors, avagacestat (Funded by Bristol-Myers Squibb; ClinicalTrials.gov identifier: NCT00890890) [9] and semagacestat (Funded by Eli Lilly; ClinicalTrials.gov identifier: NCT00594568) [10], have not displayed statistically significant benefits. Monoclonal antibodies bapineuzumab (Funded by Janssen Alzheimer Immunotherapy and Pfizer; ClinicalTrials.gov identifiers: NCT00575055, NCT00574132) [11], solanezumab (Funded by Eli Lilly; ClinicalTrials.gov identifier: NCT01900665) [12] and Gantenerumab (Funded by Roche; ClinicalTrials.gov identifier: NCT01224106) could not perform satisfactorily in Phase II and III clinical trials [8,13]. Recently, pharmaceutical giant Biogen and its Japanese partner Eisai announced that a major drug candidate (aducanumab) targeting Aβ failed in Phase III clinical trials (ClinicalTrials.gov identifiers: NCT01677572, NCT02477800, NCT02484547) [14]. However, aducanumab is now being re-considered by Biogen. Despite the disappointing track record in clinical trials of drugs that target Aβ, the amyloid hypothesis is the most widely accepted mechanism for AD.

**Figure 1:**
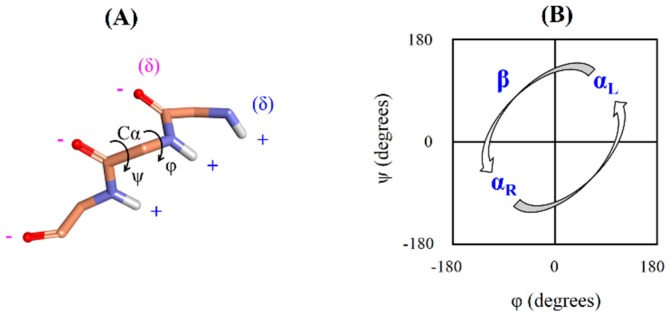
(**A**) The α-strand structure. Backbone atoms of the α-strand forming residues are displayed by the stick model. Side chains and non-polar hydrogen atoms are not shown for the sake of clarity. Alignment of main chain carbonyls on one side of the α-strand and the amide NH groups on the other side leads to two edges with opposite charges. Phi (φ) represents C-N-Cα-C dihedral angle and Psi (ψ) represents N-Cα-C-N dihedral angle. (**B**) Ramachandran or (φ,ψ) plot. The α_R_ indicates right-handed helical region, α_L_ indicates left-handed helical region and β indicates β-sheet region. The α-strand consists of successive residues with alternating dihedrals in the α_R_ and α_L_ regions.

Aβ is intrinsically disordered protein, but how it is transformed into the highly organized fibrils remains unclear. Previous studies demonstrated that Aβ fibrillogenesis is a nucleation-dependent polymerization process, which proceeds from soluble monomeric peptides through nonfibrillar oligomeric species to insoluble cross β-sheet fibrils. According to the nucleation-dependent mechanism, disordered monomer assembles into fibril nucleus through primary nucleation. This nucleus is elongated to form a mature fibril. Another fibril nucleus is formed on the surface of mature fibril through secondary nucleation that dissociates in the final step and participates in the polymerization process [4]. Recently, experimental studies have confirmed that the α-sheet structure in Aβ drives aggregation and toxicity in AD [15]. Different techniques were used to demonstrate that Aβ oligomers adopt the α-sheet structure. The A11 amyloid-oligomer-specific antibody recognized the α-sheet structure. Designed anti-α-sheet peptides inhibited Aβ aggregation and blocked toxicity in in vitro and in vivo experiments [15,16,17]. These peptides specifically recognized and neutralized the toxic, soluble Aβ oligomers in animal models of AD [15]. The α-sheet is an atypical secondary structure that is proposed as a probable intermediate state in the amyloid fibril formation based on molecular dynamics (MD) simulations of several amyloid proteins [18,19,20,21]. Unlike α-helix and β-sheet structures, dihedrals (φ,ψ) of α-sheet residues are not restricted to a specific region of the Ramachandran plot. The α-sheet is formed by bifurcated hydrogen bonding between adjacent α-strands. The α-strand/α-sheet structure comprises of successive residues with alternating dihedrals in the right-handed (α_R_) and left-handed (α_L_) helical regions. In the α-strand/α-sheet structure, main chain carbonyls are aligned on one side of the strand while the amide NH groups are aligned on the other side. Due to this alignment, the α-strand/α-sheet structure has two complementarily charged interfaces. One edge of the α-strand/α-sheet structure is negatively charged while the opposite edge is positively charged (Figure 1). The α-strand/α-sheet structures facilitate self-assembly/aggregation as a result of attractive forces between the interfaces with opposite charges. The α-sheet is reported to be the toxic intermediate structure responsible for the aggregation of various amyloid proteins [18,19,20,21]. Furthermore, several designed anti-α-sheet peptides are found to inhibit aggregation/fibrillation of various amyloid proteins and associated cytotoxicity [15,16,17,22,23].

Previously, our work on Parkinson’s disease protein α-synuclein revealed that this amyloid protein adopts the α-strand/α-sheet structure within the region that plays a crucial role in the aggregation and cytotoxicity. We proposed a novel α-strand/α-sheet based aggregation mechanism for α-synuclein and discussed strategy for designing aggregation inhibitors [21]. The latest experimental studies revealed that Aβ adopts the α-sheet structure in oligomers [15]. However, several important issues remain to be resolved. These include how an α-sheet structure is formed in Aβ; which Aβ residues are involved in the formation of this unique secondary structure; what is the effect of various factors such as temperature, pH, and point mutations on the occurrence of this structure; and how α-sheet contributes to the Aβ aggregation. In the present study, we have explained these critical issues through all-atom MD simulations on Aβ structure under different conditions. This study provides insight into the (not yet well-understood) aggregation mechanism of intrinsically disordered Aβ protein.

## 2. Results

Previous experimental studies provided evidence that Aβ adopts the α-sheet structure in oligomers and this unique secondary structure drives its aggregation and toxicity in AD [15]. The α-sheet is formed by hydrogen bonding between adjacent α-strands. In the present study, we have investigated the possibility of the formation of an α-strand structure in the Aβ_1–42_ monomer. The α-strand/α-sheet structure has been observed in several amyloid proteins earlier within 50 ns MD simulations [18,21,24,25,26]. Accordingly, 50 ns MD simulations were carried out on different monomeric structures of Aβ_1–42_ under various conditions.

### 2.1. Occurrence of α-Strand Structure in Region 24–26

**Figure 2:**
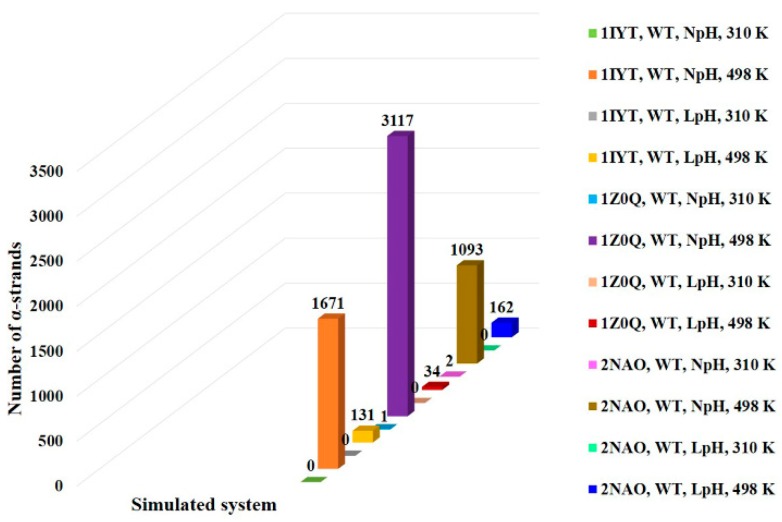
Comparison of the number of α-strands observed in region 24–26 for the simulated systems. Simulations at high temperature (498 K) showed a high number of α-strands as compared to the physiological temperature (310 K) for all the simulated PDBs. LpH simulations displayed a lower number of α-strands than NpH simulations.

MD simulations were performed on PDB 1IYT, PDB 1Z0Q, and PDB 2NAO. As shown in Figure 2, simulations at high temperature (498 K) showed a high number of α-strands in region 24–26 (VGS) for all the PDBs. However, a significant number of α-strands could not be observed in region 24–26 at physiological temperature (310 K) during the simulation time of 50 ns. The folding or unfolding of a protein may need long simulation time. Previous studies suggested that such transitions are accelerated by nonphysical temperatures [18,21,27]. This could be the reason for observing a high number of α-strands at 498 K but not at 310 K during the 50 ns simulation time. At 498 K and NpH, PDB 1IYT, PDB 1Z0Q, and PDB 2NAO displayed 1671, 3117, and 1093 α-strands (α_R_α_L_α_R_), respectively, for region 24–26 (VGS). Under the same condition, these PDBs showed 1255, 641, and 24 α-strands (α_R_α_L_α_R_α_L_), respectively, for region 24–27 (VGSN). Furthermore, they showed 455, 465, and 3 α-strands (α_R_α_L_α_R_α_L_α_R_), respectively, for region 24–28 (VGSNK). This demonstrates the possibility of extension of the α-strand region 24–26 (VGS). Several studies have reported the importance of region 23–28 in Aβ fibril formation [28,29,30]. Accordingly, the occurrence of α-strand in region 24–26 may have substantial importance.

Simulations were conducted at the LpH in addition to the NpH, to study the pH effect on the α-strand occurrence in Aβ_1–42_ peptide. As can be seen in Figure 2, the LpH condition reduced the number of α-strands for all the PDBs. At 498 K and LpH, PDB 1IYT, PDB 1Z0Q, and PDB 2NAO displayed 131, 34, and 162 α-strands, respectively. The Aβ peptide fibrillation is a complex process. It has been reported that aggregation and oligomerization of Aβ_1–42_ are strongly dependent on solution pH. Slightly acidic conditions usually increases the aggregation and fibrillation rate of Aβ_1–42_ in bulk experiments [31,32]. Our simulations results are consistent with these reports.

### 2.2. Transition of Dihedrals

**Figure 3:**
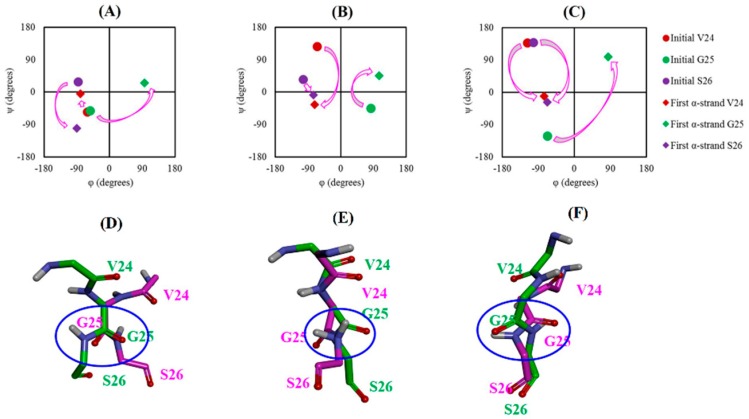
Transition of dihedrals (φ,ψ) for α-strand formation: (**A**) System 4 (1IYT, WT, NpH, 498 K), (**B**) System 5 (1Z0Q, WT, NpH, 498 K) and (**C**) System 6 (2NAO, WT, NpH, 498 K). Initial and first α-strand represents dihedrals of the specific residue in the initial PDB structure and first α-strand structure observed during MD simulation, respectively. Peptide-plane flipping: (**D**) System 4 (1IYT, WT, NpH, 498 K), (**E**) System 5 (1Z0Q, WT, NpH, 498 K) and (**F**) System 6 (2NAO, WT, NpH, 498 K). Backbone atoms of the α-strand forming residues are shown by the stick model. Side chains and non-polar hydrogen atoms are not shown for the sake of clarity. Initial conformation of the residues at the beginning of production run is displayed by the pink stick model whereas the first α-strand conformation is shown by the green stick model. Flip of the CO-NH plane is highlighted by the blue circle.

Figure 3A–C shows the dihedral transition of residues 24–26. In the case of PDB 1IYT, G25 displayed a transition from α_R_ to α_L_ region while S26 demonstrated a transition from β to α_R_ region for the formation of the first α-strand structure. For PDB 1Z0Q, V24 and S26 underwent a transition from β to α_R_ region. Besides, G25 showed a transition from its initial position to the α_L_ region. V24 and S26 of PDB 2NAO showed a transition similar to PDB 1Z0Q. However, G25 of PDB 2NAO displayed a transition similar to PDB 1IYT. These sequential transitions of dihedral angles led to the formation of the first α-strand (α_R_α_L_α_R_) structure.

The transition of the backbone dihedrals from different regions of the Ramachandran plot to the α-strand region involves rotation of the peptide-plane. Details of the peptide-plane phenomenon are provided in the previous research papers [33,34]. This phenomenon is too slow at the physiological temperature and thus requires long MD simulations [19,21]. However, high temperature accelerates such transitions. Furthermore, it is known that the peptide-plane flips back if the environment is nonconductive to the transition [19,21]. Our simulation results are in agreement with these observations. We observed a significant number of α-strands at 498 K but not at 310 K during the simulation time of 50 ns. Also, the peptide-plane flipped back and forth throughout the MD simulation. Initial and first α-strand conformation of residues 24–26 was superimposed to visualize the peptide-plane flip. Figure 3D–F shows that peptide-plane of G25–S26 underwent flipping to form the α-strand structure.

### 2.3. Effect of Mutation on Occurrence of α-Strand Structure in Region 24–26

**Figure 4:**
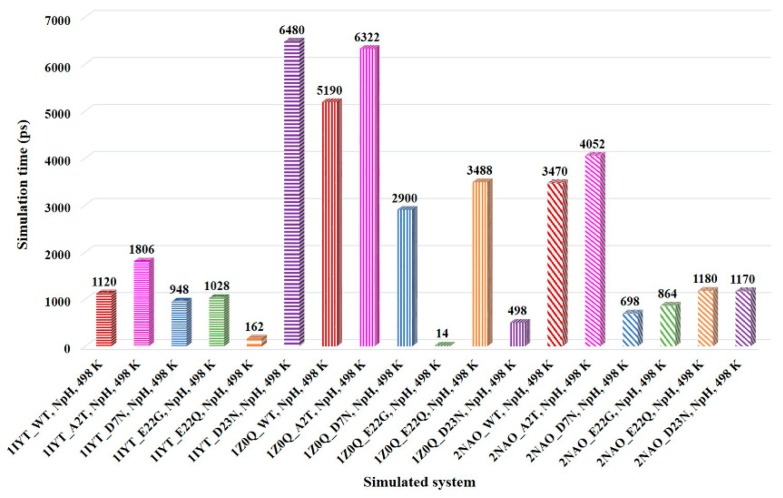
Comparison of the α-strand appearance time in region 24–26 for the simulated systems. WT, A2T, D7N, E22G, E22Q, and D23N systems are represented by red, pink, blue, green, orange, and purple bars, respectively. Except for the systems with A2T mutation and the 1IYT_ D23N system, mutant systems showed an α-strand structure in the shorter simulation time as compared to the WT systems in all the simulated PDBs.

A number of mutations are associated with familial AD. These mutations are reported to influence the aggregation and fibrillation of Aβ peptide. We performed MD simulations on several mutants which are linked to AD, such as A2T, D7N (Tottori), E22G (Arctic), E22Q (Dutch), and D23N (Iowa), to examine their influence on the α-strand formation [5,35]. Furthermore, in the present study, we designed a G25P mutant and carried out MD simulation on this mutant. The amino acid residue P is known as an inducer of the turn structure but it is rarely present in the β-sheet structure. Systems with mutants related to AD showed α-strand structure in region 24–26 similar to the WT systems at 498 K. However, except for the AD protective mutation A2T systems [35], α-strand appeared faster in the mutant systems than WT systems for all the simulated PDBs. This could be the possible reason for enhanced Aβ_1–42_ aggregation and fibrillation in D7N, E22G, E22Q, and D23N mutants [5]. In accordance with WT simulations, a significant number of α-strands could not be observed at 310 K for mutant systems. Comparison of the α-strand appearance time in region 24–26 of the Aβ_1–42_ peptide is shown in Figure 4. It can be seen that systems with D7N, E22G, and E22Q mutations displayed an α-strand structure in the shorter simulation time as compared to the WT systems in all the simulated PDBs. Except for the PDB 1IYT, systems with D23N mutation showed similar patterns as observed for the D7N, E22G, and E22Q mutations. As compared to WT and other mutant systems, protective mutation A2T delayed the formation of the α-strand structure. This is in agreement with the previous studies which reported that A2T mutation lowers the aggregation propensity of Aβ peptide [25,26,35]. In the case of the designed G25P mutant, replacement of G by P residue led to loss of the α-strand structure. All the simulated systems with G25P mutation failed to display an α-strand structure in region 24–26 at both physiological (310 K) as well as high (498 K) temperatures. Another research group studied the effect of this point mutation experimentally in rat primary neurons [36]. They found that G25P mutation not only inhibits the aggregation of Aβ_1–42_ but also prevents the neurotoxicity induced by this peptide. Furthermore, it suppresses the formation of the toxic conformer of Aβ_1–42_. Consequently, the occurrence of α-strand in region 24–26 of Aβ_1–42_ peptides may have substantial importance.

### 2.4. PDB Search for α-Strand Structure with VGS Sequence

Region 24–26 (VGS) of Aβ_1–42_ adopted the α-strand structure in the MD simulations under different conditions. Experimentally determined structures from the PDB were searched to confirm whether this identified sequence exists as an α-strand structure in the already known protein structures. Similar to our simulation results, 207 PDBs showed 464 α-strand (α_R_α_L_α_R_) structures for the VGS sequence. These PDBs included 178 X-ray crystal structures and 29 NMR structures. The number of α-strand (α_R_α_L_α_R_) structures in the crystal and NMR structures were 349 and 115, respectively. These experimental structures support the computational observation of this study. In the available Aβ_1–42_ PDBs, no α-strand structure was observed in region 24–26. Therefore, the present simulation work provides new structural insights about the Aβ_1–42_ peptide. Furthermore, this is in agreement with previous studies wherein α-strand/α-sheet was observed as a partially folded intermediate structure during MD simulations of several amyloid proteins but not in their available experimental structures [18,21,24,37,38].

## 3. Discussion

Despite tremendous efforts, there is still no curative treatment available for AD. Different therapeutic approaches are being explored for treating AD. All these approaches are in preclinical research stages and their therapeutic efficiency remains unclear [39,40]. According to the amyloid hypothesis, amyloid plaques comprising of Aβ is one of the hallmarks of AD. However, the structure of pathological aggregates and the Aβ aggregation mechanism still remains obscure. Aβ is a 38 to 43 amino acid-long peptide derived by the proteolytic cleavage of the amyloid precursor protein (APP) by β- and γ-secretases [4,41]. Aβ_1–40_ and Aβ_1–42_ are the two predominant alloforms of the Aβ peptide. The primary structure of Aβ can be divided into three regions, namely, the N-terminal (residues 1–16), the central region (residues 17–29), and the C-terminal (residues 30–40/42) [4]. Sequences of both Aβ forms are identical except the last two C-terminus residues of Aβ_1–42_. The Aβ_1–42_ is the dominant species in amyloid plaques [42].

The amyloid hypothesis has gone through many changes over the decades, mostly regarding the type of Aβ believed to cause AD: firstly, this was the amyloid plaque, afterwards, increased concentrations of Aβ_1–42_, then an increased Aβ_1–42_:Aβ_1–40_ ratio, and lastly oligomeric Aβ. Mounting evidence accumulated over the last 20 years suggests that soluble Aβ oligomers rather than insoluble Aβ fibrils initiate synapse failure and memory impairment [43]. Currently, there is intense interest in elucidating the structures of Aβ oligomers. Several types of Aβ oligomers of different sizes and shapes have been reported. These structures differ not only in aggregation states but also in their toxic effects [4,43]. Fibrils equivalently to their precursors (oligomers) and protofibrils exhibit polymorphism. Several fibril structures including U-shaped and S-shaped structures have been reported [4,44]. Biophysical techniques provide a limited understanding of the aggregated Aβ species. It is extremely challenging to obtain atomic resolution structures of oligomers [4]. Advance understanding of oligomer structures and underlying aggregation mechanism is crucial for the development of novel AD treatments.

Recent experimental studies confirmed that the α-sheet structure in Aβ drives aggregation and toxicity in AD [15]. The α-sheet is an atypical secondary structure observed in several other amyloid proteins. Transthyretin [24], lysozyme [45], β2-microglobulin [37], polyglutamine [38], and α-synuclein [21] adopted the α-sheet structure in the MD simulations. Pauling and Corey firstly proposed this structure as a pleated sheet in 1951 [46]. Many X-ray crystallography and NMR structures in the PDB contain α-strand/α-sheet structures [19,21]. Experimentally determined PDB structures have confirmed the existence of α-strand/α-sheet and provided evidence that they are not just computational or theoretical artifacts. The α-sheet is reported to be the toxic intermediate structure accountable for aggregation of several amyloid proteins [18,19,20]. Additionally, a number of designed anti-α-sheet peptides are found to inhibit aggregation and toxicity of various amyloid proteins [15,16,17,22,23,47]. Although, it is known that the α-sheet structure in Aβ drives aggregation and toxicity in AD [15], the crucial details of α-sheet formation in Aβ are not known. These include how α-sheet is formed in Aβ, which region and residues of Aβ adopt this structure, what is the effect of various factors such as temperature, pH, and point mutations on the occurrence of this structure and how α-sheet contributes to Aβ aggregation. In the present work, we attempted to address these questions through MD simulation studies. Three different WT structures of full-length human Aβ_1–42_ were used for MD simulations to avoid bias in the initial conformation. These structures included Aβ_1–42_ monomer structure in an apolar microenvironment, Aβ_1–42_ monomer structure in HFIP/aqueous mixture, and amyloid fibril structure of disease-relevant Aβ_1–42_. Several mutations are associated with AD such as A2T, D7N, E22G, E22Q, and D23N [5,35]. We computationally mutated WT structures to produce mutant structures. In accordance with former studies on α-strand/α-sheet structure [18,21], MD simulations were carried out on WT and mutant structures at high temperature (498 K) in addition to the physiological temperature (310 K) for 50 ns. Simulations were performed at NpH and LpH to investigate the effect of pH.

MD simulations under various conditions revealed that intrinsically disordered Aβ_1–42_ monomer adopts the α-strand structure in the central region by peptide-plane flipping. Simulations at high temperature showed a high number of α-strands as compared to physiological temperature during the simulation time of 50 ns. This is in agreement with previous studies which reported that elevated or nonphysical temperatures accelerate protein folding/unfolding process without altering the pathway [18,21,24]. The peptide-plane flipping phenomenon is too slow at physiological temperature and thus requires long MD simulations [19,21]. However, high temperatures accelerate such transitions. The LpH condition displayed a low number of α-strands as compared to NpH condition. This result is consistent with former studies which reported that aggregation of Aβ_1–42_ is strongly dependent on solution pH and acidic condition decreases its aggregation [31,32]. The α-strand structure appeared faster in the D7N, E22G, E22Q, and D23N mutant systems than WT systems. This could be the possible reason for enhanced Aβ_1–42_ aggregation in these mutants [5]. As compared to WT and other mutant systems, protective mutation A2T delayed the formation of the α-strand structure. Previous studies reported that A2T mutation lowers the aggregation propensity of Aβ peptide [25,26,35]. Delayed formation of the α-strand structure could possibly explain the delayed and low aggregation of the A2T mutant.

Residues 24–26 (VGS) of the central region were found to be involved in the formation of the α-strand structure. Furthermore, results showed the possibility of extension of α-strand structure from region 24–26 (VGS) to region 24–28 (VGSNK). We mutated central α-strand residue from G to P (G25P) and carried out MD simulations on this mutant. This mutation leads to the loss of α-strand structure. This is in agreement with an earlier experimental study which reported that the G25P mutation not only inhibits the aggregation of Aβ_1–42_ but also prevents the neurotoxicity induced by this peptide [36]. A number of previous studies have reported the significance of region 23–28 in Aβ fibril formation [28,29,30]. Accordingly, the α-strand structure formed by residues 24–26 may have substantial importance in Aβ aggregation.

**Figure 5:**
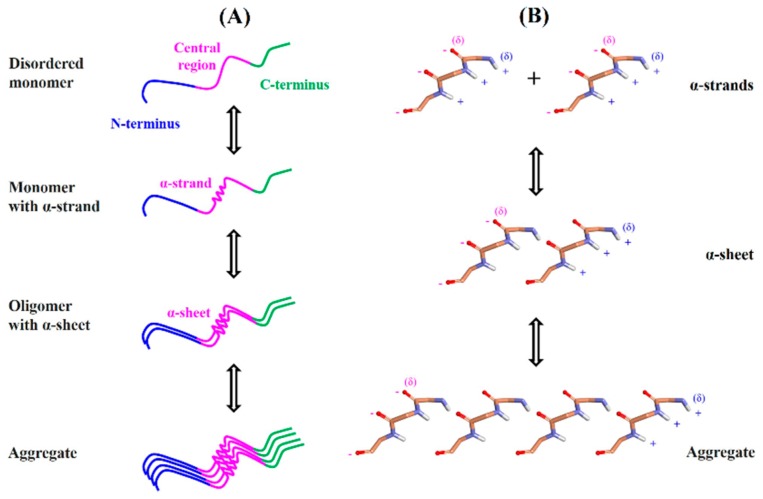
Proposed aggregation mechanism. (**A**) Schematic representation showing α-strand/ α-sheet formation during Aβ_1–42_ aggregation process. N-terminal, central region, and C-terminal are indicated by blue, pink, and green colors, respectively. Central region (residues 24–26) of the monomer adopts the α-strand conformation occasionally. Hydrogen bonding between the α-strands leads to the formation of α-sheet structure in the oligomers. The α-sheet structure acts as a nucleus that initiates the aggregation process. Elongation of nucleus through the incorporation of further Aβ_1–42_ molecules form a mature fibril. (**B**) Alignment of carbonyl and amino groups generates two complementarily charged interfaces in the α-sheet structure. Attractive forces between the interfaces with opposite charges facilitate the aggregation of Aβ_1–42_. Negative and positive partial charges on the interface are shown by red and blue colors, respectively.

Here, we propose a novel α-strand/α-sheet-based aggregation mechanism of Aβ_1–42_. According to the proposed mechanism (Figure 5), central region residues 24–26 (VGS) of the Aβ_1–42_ monomer adopts an α-strand structure. This structure proceeds to the α-sheet structure in the oligomers through hydrogen bonding. The α-sheet acts as a nucleus for the oligomerization and fibrillation. Two interfaces with opposite charges in the α-sheet structure, due to the alignment of carbonyl and amino groups, facilitates self-association into soluble oligomeric amyloid protofibrils. The protofibrils undergo a transition from the toxic soluble phase to the insoluble, more highly ordered amyloid fibrils composed of both parallel and antiparallel β-sheets. It involves a transition from α-sheet to β-sheet via peptide-plane flipping. Amyloid fibrils are structurally very heterogeneous. It may be possible that both α-sheet and β-sheet are present in the mature fibril depending on the sequence and conditions [19]. This hypothesis is in agreement with the known nucleation-dependent Aβ aggregation mechanism [4,48]. Furthermore, our proposed mechanism is supported by the recent experimental findings which suggested that the α-sheet structure in Aβ drives aggregation and toxicity in AD [15]. The identified α-strand/α-sheet-forming region (residue 24–26) of Aβ_1–42_ could be targeted by designed anti-α-sheet peptides.

## 4. Materials and Methods

### 4.1. Structures of Human Aβ_1–42_ and Mutants

**Figure 6:**
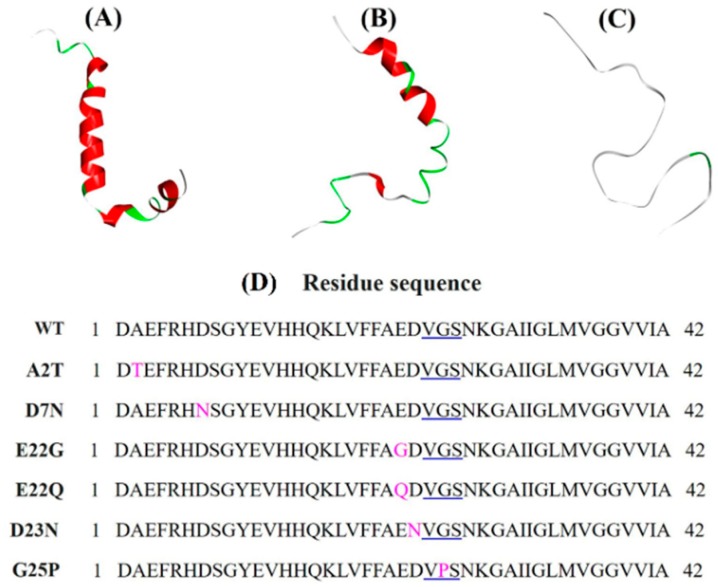
MD simulations were performed on three different structures of Aβ_1–42_ to avoid bias in the initial conformation: (**A**) PDB 1IYT, chain A, model 5 (NMR structure in an apolar microenvironment); (**B**) PDB 1Z0Q, chain A, model 15 (NMR structure in HFIP/aqueous mixture); (**C**) PDB 2NAO, chain B, model 5 (NMR amyloid fibril structure of disease-relevant Aβ_1–42_). (**D**) Residue sequences of the WT Aβ_1–42_ and its mutants. Residues 24–26 are underlined in blue. Mutated residues are marked in pink color.

A number of Aβ_1–42_ structures are available in the RCSB protein data bank (PDB, http://www.rcsb.org/). An X-ray crystal structure for the full Aβ_1–42_ sequence does not exist so far, but monomers, as well as fibril nuclear magnetic resonance (NMR) structures, are available for full-length Aβ_1–42_. Three different wild-type (WT) NMR structures of full-length human Aβ_1–42_ were retrieved from PDB for the MD simulation to avoid bias in the initial conformation. These structures included PDB 1IYT (Aβ_1–42_ monomer structure in an apolar microenvironment) [49], PDB 1Z0Q (Aβ_1–42_ monomer structure in hexafluoroisopropanol (HFIP)/aqueous mixture) [50], and PDB 2NAO (amyloid fibril structure of disease-relevant Aβ_1–42_) [51]. Several NMR models were available for these PDBs. Three NMR models were selected randomly for the present study. Model 5 of PDB 1IYT (chain: A, total models: 10), model 15 of PDB 1Z0Q (chain: A, total models: 30), and model 5, chain B of PDB 2NAO (chains: A-F, total models: 10) were chosen for the MD simulations (Figure 6A–C). Discovery Studio 2018 (BIOVIA, Dassault Systèmes, San Diego, CA, USA) was used for extracting the models. These WT NMR structures were computationally mutated to produce mutant structures. Point mutations were created using the mutagenesis-wizard of the PyMol package (The PyMOL Molecular Graphics System, Schrödinger, LLC). Residue sequences of the WT Aβ_1–42_ and its mutants are provided in Figure 6D.

### 4.2. Molecular Dynamics Simulation

GROMACS 5.1.3 package [52] with CHARMM36m force field [53] was employed to perform MD simulations. PDBs 1IYT [49], 1Z0Q [50] and 2NAO [51] were used for the simulations. Elevated temperature accelerates protein unfolding without altering the unfolding pathway [27]. MD simulations have been performed at 498 K to accelerate the folding process in the previous studies on several amyloid proteins [18,21]. In accordance with these works, MD simulations were performed at high temperature (498 K) in addition to the physiological temperature (310 K). MD simulations were carried out at neutral pH (NpH) and low pH (LpH: protonated His, Asp, and Glu) [18,24] to study the effect of pH. Protein was solvated in a cubic box of TIP3P water molecules. An appropriate number of counter-ions (Na^+^ and Cl^−^) were added to the box depending upon the protonation state of the protein. The energy of the system was minimized using 50,000 steps of the steepest descent algorithm. The system was equilibrated in two phases. In the first phase, equilibration was conducted for 0.1 nanosecond (ns) under an NVT ensemble (constant number of particles, volume, and temperature). Temperature was maintained with a V-rescale thermostat. In the second phase, equilibration was conducted for 1 ns under an NPT ensemble (constant number of particles, pressure and temperature). Pressure was maintained at 1 bar using a Parrinello–Rahman barostat. Finally, a production run was carried out for 50 ns under periodic boundary conditions and coordinate trajectories were recorded every 2 ps. Bond lengths were constrained using the linear constraint solver (LINCS) algorithm. Short-range interactions were truncated at 14 Å and long-range interactions were handled with the particle mesh Ewald (PME) method. Table 1 lists the systems on which MD simulations were performed.

### 4.3. Computation of Dihedral Angles

The gmx rama module of GROMACS software [52] was used for calculating the dihedral angles. Firstly, the dihedral angles of all the residues were computed throughout the MD simulation. Then, residues that adopted α-strand conformation during the simulation were identified using the in-house R scripts. These scripts also provided the number of α-strand conformations. The α-strand consists of successive residues with alternating α_R_ and α_L_ conformations. In accordance with the former articles on α-strand [19,21], a residue was categorized in the α_R_ conformation if its dihedral angles were within −180 < φ < 0, −180 < ψ < 0 i.e., (−, −). Whereas, a residue with dihedral angles within 0 < φ < 180, 0 < ψ < 180 i.e., (+, +) was classified in the α_L_ conformation.

### 4.4. PDB Search for α-Strand Structure

All the available structures (155,160 PDBs) were downloaded from the RCSB PDB website (RCSB PDB; available online: http://ftp.rcsb.org (accessed on 13 September 2019)). The non-protein structures (10,655 PDBs) such as deoxyribonucleic acid (DNA) and ribonucleic acid (RNA) structures were removed. All the protein structures (144,505 PDBs) were searched for the α-strand structure using the in-house R scripts.

## Figures and Tables

**Table 1:** MD simulations performed in the present study. Aggregated simulation time is 1950 ns.

Simulated System	PDB, Type	Counter Ion	Temperature	pH	MD Run
1	1IYT, WT	3 Na^+^	310 K	NpH	50 ns
2	1Z0Q, WT	3 Na^+^	310 K	NpH	50 ns
3	2NAO, WT	3 Na^+^	310 K	NpH	50 ns
4	1IYT, WT	3 Na^+^	498 K	NpH	50 ns
5	1Z0Q, WT	3 Na^+^	498 K	NpH	50 ns
6	2NAO, WT	3 Na^+^	498 K	NpH	50 ns
7	1IYT, WT	6 Cl^−^	310 K	LpH	50 ns
8	1Z0Q, WT	6 Cl^−^	310 K	LpH	50 ns
9	2NAO, WT	6 Cl^−^	310 K	LpH	50 ns
10	1IYT, WT	6 Cl^−^	498 K	LpH	50 ns
11	1Z0Q, WT	6 Cl^−^	498 K	LpH	50 ns
12	2NAO, WT	6 Cl^−^	498 K	LpH	50 ns
13	1IYT, Mutant (A2T)	3 Na^+^	498 K	NpH	50 ns
14	1Z0Q, Mutant (A2T)	3 Na^+^	498 K	NpH	50 ns
15	2NAO, Mutant (A2T)	3 Na^+^	498 K	NpH	50 ns
16	1IYT, Mutant (D7N)	2 Na^+^	498 K	NpH	50 ns
17	1Z0Q, Mutant (D7N)	2 Na^+^	498 K	NpH	50 ns
18	2NAO, Mutant (D7N)	2 Na^+^	498 K	NpH	50 ns
19	1IYT, Mutant (E22G)	2 Na^+^	310 K	NpH	50 ns
20	1Z0Q, Mutant (E22G)	2 Na^+^	310 K	NpH	50 ns
21	2NAO, Mutant (E22G)	2 Na^+^	310 K	NpH	50 ns
22	1IYT, Mutant (E22G)	2 Na^+^	498 K	NpH	50 ns
23	1Z0Q, Mutant (E22G)	2 Na^+^	498 K	NpH	50 ns
24	2NAO, Mutant (E22G)	2 Na^+^	498 K	NpH	50 ns
25	1IYT, Mutant (E22Q)	2 Na^+^	310 K	NpH	50 ns
26	1Z0Q, Mutant (E22Q)	2 Na^+^	310 K	NpH	50 ns
27	2NAO, Mutant (E22Q)	2 Na^+^	310 K	NpH	50 ns
28	1IYT, Mutant (E22Q)	2 Na^+^	498 K	NpH	50 ns
29	1Z0Q, Mutant (E22Q)	2 Na^+^	498 K	NpH	50 ns
30	2NAO, Mutant (E22Q)	2 Na^+^	498 K	NpH	50 ns
31	1IYT, Mutant (D23N)	2 Na^+^	498 K	NpH	50 ns
32	1Z0Q, Mutant (D23N)	2 Na^+^	498 K	NpH	50 ns
33	2NAO, Mutant (D23N)	2 Na^+^	498 K	NpH	50 ns
34	1IYT, Mutant (G25P)	3 Na^+^	310 K	NpH	50 ns
35	1Z0Q, Mutant (G25P)	3 Na^+^	310 K	NpH	50 ns
36	2NAO, Mutant (G25P)	3 Na^+^	310 K	NpH	50 ns
37	1IYT, Mutant (G25P)	3 Na^+^	498 K	NpH	50 ns
38	1Z0Q, Mutant (G25P)	3 Na^+^	498 K	NpH	50 ns
39	2NAO, Mutant (G25P)	3 Na^+^	498 K	NpH	50 ns
